# Supplementary material for: Tailoring the Diameters of Electro-Mechanically Spun Fibers by Controlling Their Deborah Numbers
Source: Polymers (Basel). 2020 Jun 17;12(6):1358. doi: 10.3390/polym12061358 (PMC7361984; doi:10.3390/polym12061358)
Supplement: Supplementary file 1 [file polymers-12-01358-s001.pdf]

polymers

Supplementary Materials: Tailoring the Diameters of Electro-Mechanically Spun Fibers by Controlling Their Deborah Numbers

Domingo R. Flores-Hernández [1], Braulio Cardenas-Benitez [1], Sergio O. Martinez-Chapa [1], and Jaime Bonilla-Rios [1,] *

Domingo R. Flores-Hernandez, Braulio Cardenas-Benitez, Sergio O. Martinez-Chapa, and Jaime Bonilla-Rios *

Tecnologico de Monterrey, Escuela de Ingeniería y Ciencias, Ave. Eugenio Garza Sada 2501, Monterrey 64849, NL, Mexico; drflores@tec.mx (D.R.F.-H.); braulio.cardenas@uci.edu (B.C.-R.); smart@tec.mx (S.O.M.-C.)
* Correspondence: jbonilla@tec.mx; Tel.: 52-81-8358-2000 Ext. 5721

Contents

Oscillatory Tests 2
Code for Maxwell elements estimation 2
Relaxation Spectra 9

Oscillatory Tests

In this section is presented the data obtained from frequency sweeps to all the polymer solutions. All rheological tests were performed in a rotational rheometer (Physica MCR 301, Anton Paar) equipped with a cone-and-plate (CP) geometry (diameter of 24.98 mm, angle of 4.014° and truncation of 249 μm). Experiments were conducted at 25 ± 0.1°C and 24 hours after polymer solution preparation. Frequency sweeps, to determine the loss and storage modulus, were performed at an amplitude strain of %γ=20 in the linear visco-elastic regime.

Table S1. Frequency sweeps of all polymer solutions.

		Concentration of PEO=TBATFB [wt%]							
		0.25wt%		0.50wt%		0.75%		1wt%	
Meas. Pts.	Angular Frequency	G'	G''	G'	G''	G'	G''	G'	G''
	[1/s]	[Pa]							
1	0.10	0.32	1.66	1.87	6.93	5.28	13.80	11.60	19.80
2	0.16	0.48	1.96	3.60	10.90	8.75	20.30	16.60	27.80
3	0.25	0.94	3.72	5.93	16.00	11.60	25.30	22.40	36.60
4	0.40	1.32	5.13	8.25	21.20	15.40	32.70	27.50	43.90
5	0.63	1.67	6.34	10.10	25.40	19.30	39.30	33.30	51.00
6	1.00	2.10	7.96	12.30	30.50	22.70	44.30	40.20	57.60
7	1.58	2.61	9.91	14.10	35.00	26.40	48.90	48.10	63.30
8	2.51	3.23	12.30	16.20	39.60	30.30	53.30	56.60	68.00
9	3.98	3.92	15.20	18.40	44.30	34.10	57.40	64.40	72.40
10	6.31	4.66	18.40	20.40	49.00	38.10	61.40	72.50	77.40
11	10.00	5.46	22.20	21.80	53.70	42.40	65.00	81.20	80.80
12	15.80	6.30	26.70	24.90	58.40	47.20	67.40	86.60	84.30
13	25.10	7.30	33.10	27.50	61.80	49.80	68.90	92.10	86.60
14	39.80	9.44	44.50	31.70	65.60	52.80	71.70	95.90	90.30
15	63.10	11.80	57.50	34.90	69.10	56.60	76.90	102.00	99.20
16	100.00	10.90	75.30	37.60	79.60	57.80	89.70	103.00	116.00

G'=Storage Modulus; G''=Loss Modulus

Code for Maxwell elements estimation

The following algorithm was encoded in Fortan 95. It takes as incomes a number of measurements, the number of Maxwell wanted, the angular frequency, and the loss modulus. As outputs, it gives the lambda and eta from the Maxwell elements defined.

```
#include <stdio.h>
#include <stdlib.h>
#include <math.h>
#include <time.h>

#define maxN 8                    /* Maximum Maxwell elements */
#define decade 10.0               /* Consecutive lamdas ratio */
#define h 0.00000001      /* Defining small increment */
#define maxn 30                   /* Defining maximum experimental points */
#define maxit 50000               /* Maximum iterations */
#define weight 1.0                /* Weight number between 0 and 2 */
#define EPS 0.00001;      /* Maximum stopping criteria */
                                  /*10*/
```

```
#define CONST 0.5                    /* constant for minimum lamda (<1.0) */

double stop[maxN];         /* Variable stopping criteria vector */

int N;                                              /* Desired Maxwell Elements */
char resinname[20];
/*int g was supressed, no. of experimental points is counted automatically*/
/* array w is read automatically*/
/* array lossG is read automatically*/
double lamda[maxN] = { 0 };         /* Relaxation times */
double eta[maxN];            /* Relaxation Spectrum Parameters */

/* names of files to be loaded */
//char frec[] = "frec.txt";
//char gbiprime[] = "gbiprime.txt";

/* names of files where results will be saved */
//char rlambdas[] = "lambdas.txt";
//char retas[] = "etas.txt";

void nonlinear(double xdata[], double ydata[], double p[], int psize, int points, char fname3, char fname4);
double lossmodulus(const double x, const double p[]);
double r(int k);
int Ndata(char fname1[], char fname2[]);

main() {
        int i, k;

        /* User input */
        printf("\nInput name of file (resin) that you want to analyze: ");
        scanf("%s", &resinname);
        printf("\nNumber of maxwell elements?:  ");
        scanf("%d", &N);

        /* Names of files for input and output*/
        char ifrec[] = "frec.txt";
        char igbiprime[] = "gbiprime.txt";
        char ilambdas[] = "lambdas.txt";
        char ietas[] = "etas.txt";

        /* Specifying location of previous files*/

        char frec[25]; //   /resinname/frec.txt
        char gbiprime[25];
        char rlambdas[25];
        char retas[25];
        // puts resin name into address

        printf("Specifying location of previous files\n"); ////

        /* Opcion 1 */
        sprintf(frec, "./%s/frec.txt", resinname);
        sprintf(gbiprime, "./%s/gbiprime.txt", resinname);
        sprintf(rlambdas, "./%s/lambdas.txt", resinname);
        sprintf(retas, "./%s/etas.txt", resinname);
        printf("%s\n", frec); //example

        getchar();

        int ndata = Ndata(frec, gbiprime);   /* Amount of experimental points*/

        printf("\nThe number of maxwell elements is limited by the frequency range");
```

```
printf("\nand by the quality of the experimental data.");

printf("\n\nExperimental points will be loaded automatically from ");
printf("\nfiles 'freq.txt' and 'gbiprime.txt' under %s file.   Number of data: %d", resinname, ndata);

/* Creation of w and lossG as variable arrays*/
double *w, *lossG;
double bufferw, bufferG;
w = (double*)calloc(ndata, sizeof(double));
lossG = (double*)calloc(ndata, sizeof(double));

/* Opening files */
FILE *f1;
f1 = fopen(frec, "r");

FILE *f2;
f2 = fopen(gbiprime, "r");

if (f1 == NULL) {
        perror("\n\nFrecuencies file could not be opened. Press any key to exit.");
        getchar();

        exit(EXIT_FAILURE);
}
else printf("\nFrecuencies file accessed\n");

if (f2 == NULL) {
        perror("\nG biprime file could not be opened. Press any key to exit.");
        getchar();

        exit(EXIT_FAILURE);
}
else printf("\nG biprime file accessed\n");

/* Loading Data */
printf("\nLoading data... Press any key.\n");

for (k = 0; k < ndata; k++) {
        fscanf(f1, "%lf", &bufferw);
        w[k] = bufferw;
}

printf("\nThe frecuencies loaded are:\n");
for (i = 0; i < ndata; i++)   {
        printf("%lf \n", w[i]);
}

for (k = 0; k < ndata; k++) {
        fscanf(f2, "%lf", &bufferG);
        lossG[k] = bufferG;
}

printf("\nThe G biprimes loaded are:\n");
for (i = 0; i < ndata; i++)   {
        printf("%lf \n", lossG[i]);
}

/* Closing files */
fclose(f1);
fclose(f2);

printf("\nPress any key to proceed.\n");
```

```
	getchar();

	stop[0] = EPS;	/* Stopping criteria determination for each parameter */
	for (i = 1; i <= N - 1; i++) {
		stop[i] = stop[i - 1] * 10.0;
		if (i == N - 2)
			stop[i] = stop[i - 1];
	}

	lamda[0] = CONST / w[ndata - 1];			/* Relaxation times determination */
	for (k = 1; k <= N - 1; k++)
		lamda[k] = lamda[k - 1] * decade;

	eta[0] = lamda[N - 1];			/* Initial Guesses */
	for (k = 1; k <= N - 1; k++)
		eta[k] = (eta[k - 1]) * (r(k) + 0.5);

	nonlinear(w, lossG, eta, N, ndata, rlambdas, retas);			/* Call main function */
	return 0;
}    /* Main end */


/* FUNCTIONS DEFINITIOS */
double lossmodulus(const double x, const double p[maxN])

{
	int k;
	double loss = 0;

	for (k = 0; k <= N - 1; k++)
		loss += (p[k] * x) / (1.0 + pow(lamda[k] * x, 2.0));
	return loss;
}

double r(int k)

{
	static int z[maxN];
	int a = 0;
	int b = 3011;  /* A large prime number (2^31-1)        */
	int c = 13;    /* An integer between 2,3,..,b-1 (7^5) */
	double result;

	/* Ri = x + (float)(rand()/RAND_MAX)*(y-x)          */
	/* Generates random numbers between x and y         */
	/*      with gaps of 0.1 between them             */

	srand(time(NULL));
	/* Seed can be any number between 1, 2,..,b-1 */
	z[0] = 1 + (rand()) % (b - 1);

	z[k] = (a + c*z[k - 1]) % b;
	result = ((double)(z[k])) / ((double)(b));
	return result;		/* random number between 0 and 1 */
}

void nonlinear(double xdata[maxn], double ydata[maxn], double p[maxN], int psize, int points, char
fname3[12], char fname4[9])
{
	double J[maxn][maxN];		/* Jacobian Matrix */
	double Jt[maxN][maxn];		/* Transpose Jacobian Matrix */
	double JtJ[maxN][maxN];
```

```
double E[maxn];                    /* Difference vector    */
double JtE[maxN];
double A[maxN];                                        /* Approximation vector */
int i, j, k, l;
int iter, iter2;
double temp = 0;
double eval1, eval2;
double errorJ = 0;
double errorN = 0;
double sum = 0;
double dummy, old, pnew;
double sentinel, sentinel2;
double a, b;
char key;
double SSmean, SS, spread, rr, residual[maxn];

for (i = 0; i <= psize - 1; i++)
        A[i] = p[i];            /* first approximation */

sentinel = 0;
iter = 0;
while ((iter < maxit) && (sentinel == 0))  {
        sentinel = 1;
        iter += 1;

        /* Fill Jacobian Matrix */
        for (i = 0; i <= points - 1; i++)
                for (k = 0; k <= psize - 1; k++) {
                        temp = p[k];
                        p[k] += h;
                        eval1 = lossmodulus(xdata[i], p);
                        p[k] = temp;
                        eval2 = lossmodulus(xdata[i], p);
                        J[i][k] = (eval1 - eval2) / h;
                }

        /* Fill Difference Matrix */
        for (i = 0; i <= points - 1; i++)
                E[i] = ydata[i] - lossmodulus(xdata[i], p);

        /* Fill tranposed Jacobian Matriz */
        for (i = 0; i <= psize - 1; i++)
                for (k = 0; k <= points - 1; k++)
                        Jt[i][k] = J[k][i];

        for (i = 0; i <= psize - 1; i++)
                for (k = 0; k <= psize - 1; k++) {
                        for (l = 0; l <= points - 1; l++)
                                sum += Jt[i][l] * J[l][k];
                        JtJ[i][k] = sum;
                        sum = 0;
                }

        for (i = 0; i <= psize - 1; i++) {
                for (l = 0; l <= points - 1; l++)
                        sum += Jt[i][l] * E[l];
                JtE[i] = sum;
                sum = 0;
        }

        /* Gauss-Seidel Method for solving matrix system */
        sentinel2 = 0;
```

```
		iter2 = 0;
		while ((iter2 < maxit) && (sentinel2 == 0)) {
			sentinel2 = 1;
			iter2 += 1;
			for (i = 0; i <= psize - 1; i++) {
				old = A[i];
				sum = JtE[i];
				for (j = 0; j <= psize - 1; j++)
					if (i != j)
						sum = (sum)-(JtJ[i][j] * A[j]);
				sum = sum / JtJ[i][i];
				A[i] = weight * sum + (1 - weight) * old;
				if ((sentinel2 == 1) && (A[i] != 0.0)) {
					errorJ = fabs((A[i] - old) / (A[i]));
					if (errorJ > stop[i])
						sentinel2 = 0;
				}
			}
			printf("\n%d-%d", iter, iter2);
		} /* while #2 end */

		for (k = 0; k <= psize - 1; k++) {
			pnew = p[k] + A[k];		/* New Approximation */
			if ((sentinel == 1) && (pnew != 0.0)) {
				errorN = fabs((pnew - p[k]) / (pnew));
				if (errorN > stop[k])
					sentinel = 0;
			}
			p[k] = pnew;
		}
	} /* while #1 end */

	/* eta[N] correction */
	b = (log(eta[N - 3] / lamda[N - 3]) - log(eta[N - 2] / lamda[N - 2])) /
		(log(lamda[N - 3]) - log(lamda[N - 2]));
	a = exp(log(eta[N - 2] / lamda[N - 2]) - b*log(lamda[N - 2]));
	eta[N - 1] = lamda[N - 1] * (a*pow(lamda[N - 1], b));

	/* Opening files for saving results */
	FILE *f3;
	f3 = fopen(fname3, "w");

	FILE *f4;
	f4 = fopen(fname4, "w");

	if (f3 == NULL) {
		perror("\n\nFile for saving lambdas could not be opened");
		getchar();
		exit(EXIT_FAILURE);
	}
	else printf("\nFile for saving lambdas accessed %s:\n", fname3);

	if (f4 == NULL) {
		perror("\nFile for saving etas could not be opened");
		getchar();
		exit(EXIT_FAILURE);
	}
	else printf("\nFile for saving etas accessed %s:\n", fname4);

	/* print and save results */
	printf("\n\nThe Discrete Relaxation Spectrum \n");
	for (k = 0; k <= N - 1; k++) {
```

```
		if (p[k] > 0.0) {
			printf("\n lamda #%d = %f   eta #%d = %f ", k, lamda[k], k, p[k]);
			fprintf(f3, "%lf\n", lamda[k]);
			fprintf(f4, "%lf\n", p[k]);
		}
		else {
			printf("\n Warning! Ill-posed problem!");
			printf("\n The frequency range is not enough to specify %d relaxation points.", N);
			printf("\n It is recommended to start again the program and specify a smaller N.");
		}
	}

	/* Closing save files*/
	fclose(f3);
	fclose(f4);

	printf("\n Press any key to see statistical results:");
	scanf("%c", &key);
	printf("\n");
	sum = 0;
	for (i = 0; i <= points - 1; i++)
		sum += ydata[i];
	SSmean = 0;
	SS = 0;
	for (i = 0; i <= points - 1; i++) {
		SSmean += pow((ydata[i] - sum / ((double)(points))), 2.0);
		SS += pow((ydata[i] - lossmodulus(xdata[i], p)), 2.0);
		residual[i] = fabs((ydata[i] - lossmodulus(xdata[i], p)) / ydata[i] * 100.0);
	}
	spread = pow((SS / ((double)(points - N))), 1.0 / 2.0);
	rr = (SSmean - SS) / SSmean;
	for (i = 0; i <= points - 1; i++)
		printf("\n Residual %d = %f", i, residual[i]);
	printf("\n SS = %f \n SSmean = %f \n Spread = %f \n R^2 = %f \n", SS, SSmean, spread, rr);
	printf("\nPress any key to continue");
	scanf("%c", &key);

} /* Nonlinear Function End */

int Ndata(char fname1[9], char fname2[13] ) {
	int i = 0, j = 0;
	double buffer, buffer2;

	FILE *f1;
	f1 = fopen(fname1, "r");

	FILE *f2;
	f2 = fopen(fname2, "r");

	if (f1 == NULL) {
		perror("\nFrecuencies file could not be opened");
		getchar();
		exit(EXIT_FAILURE);
	}
	else printf("\nFrecuencies file accessed\n");

	//Contador de datos en el archivo
	while (fscanf(f1, "%lf", &buffer) != EOF) {
		i++;
	}
	printf("\nNumber of frecuencies to process %d\n", i);
```

```
    if (f2 == NULL) {
        perror("\nG biprime file could not be opened");
        getchar();
        exit(EXIT_FAILURE);
    }
    else printf("\nG biprime file accessed\n");


    //Contador de datos en el archivo
    while (fscanf(f2, "%lf", &buffer2) != EOF) {
        j++;
    }
    printf("\nNumber of Gbiprimes to process %d\n", j);

    if (j == i) {
        printf("\nNumber of frecuencies is equal to number of G's. OK.\n");
        fclose(f1);
        fclose(f2);
        return i;
    }
    else {
        perror("\nNumber of frecuencies and G's do not match. Aborting.");
        getchar();
        exit(EXIT_FAILURE);
    }
}
```

Relaxation Spectra H(λ) and $\lambda_{logmean}$

Table S2. Maxwell element components.

	Concentration of PEO=TBATFB [wt%]											
	0.25wt%			0.50wt%			0.75%			1wt%		
Maxwell Element	η_i	ai	λi [s]	η_i	ai	λi [s]	η_i	ai	λi [s]	η_i	ai	λi [s]
1	0.155	300	0.001	0.090	250	0.000	0.063	180	0.000	0.021	150	0.000
2	1.073	190	0.006	0.563	220	0.003	0.276	150	0.002	0.072	120	0.001
3	5.998	98	0.061	3.247	82	0.040	1.775	90	0.020	0.283	90	0.003
4	19.089	68	0.281	12.470	59	0.211	6.714	57	0.118	0.808	70	0.012
5	60.756	52	1.168	47.889	48	0.998	25.390	38	0.668	2.303	19	0.125
6	140.000	31	4.516	90.000	21	4.286	45.000	16	2.813	10.500	10	1.019
Log. mean			1.37			1.25			0.74			0.21
